# Improved Salt Tolerance and Metabolomics Analysis of *Synechococcus elongatus* UTEX 2973 by Overexpressing Mrp Antiporters

**DOI:** 10.3389/fbioe.2020.00500

**Published:** 2020-05-26

**Authors:** Jinyu Cui, Tao Sun, Shubin Li, Yaru Xie, Xinyu Song, Fangzhong Wang, Lei Chen, Weiwen Zhang

**Affiliations:** ^1^Laboratory of Synthetic Microbiology, School of Chemical Engineering and Technology, Tianjin University, Tianjin, China; ^2^Key Laboratory of Systems Bioengineering, Frontier Science Center for Synthetic Biology, Ministry of Education of China, Tianjin, China; ^3^Collaborative Innovation Center of Chemical Science and Engineering, Tianjin, China; ^4^Center for Biosafety Research and Strategy, Tianjin University, Tianjin, China

**Keywords:** cyanobacteria, Mrp antiporter, metabolomic analysis, salt tolerance, tolerance engineering

## Abstract

The fast-growing cyanobacterium *Synechococcus elongatus* UTEX 2973 (Syn2973) is a promising candidate for photosynthetic microbial factory. Seawater utilization is necessary for large-scale cultivation of Syn2973 in the future. However, Syn2973 is sensitive to salt stress, making it necessary to improve its salt tolerance. In this study, 21 exogenous putative transporters were individually overexpressed in Syn2973 to evaluate their effects on salt tolerance. The results showed the overexpression of three Mrp antiporters significantly improved the salt tolerance of Syn2973. Notably, overexpressing the Mrp antiporter from *Synechococcus* sp. PCC 7002 improved cell growth by 57.7% under 0.4 M NaCl condition. In addition, the metabolomics and biomass composition analyses revealed the possible mechanisms against salt stress in both Syn2973 and the genetically engineered strain. The study provides important engineering strategies to improve salt tolerance of Syn2973 and is valuable for understanding mechanisms of salt tolerance in cyanobacteria.

## Introduction

Cyanobacteria are a large group of gram-negative prokaryotes capable of taking solar energy and CO_2_ as the sole energy and carbon source for growth, respectively (Stanier and Cohen-Bazire, 1977). In recent years, the development of synthetic biology strategies and tools allowed establishment of cyanobacteria as photosynthetic “microbial cell factories” to produce renewable fuels and chemicals, as a promising alternative to traditional petroleum-based production (Gao et al., 2016b), such as ethanol, isoprene, 3-hydroxypropionic acid, and so on (Gao et al., 2012, 2016a; Wang et al., 2016). However, the productivity of cyanobacteria was still relatively lower than that of the traditional heterotrophic hosts such as *Escherichia coli* and *Saccharomyces cerevisiae*, mostly due to their relatively slow growth rates (Li et al., 2018). *Synechococcus elongatus* UTEX 2973 (hereafter Syn2973) is a recently isolated cyanobacterial strain, which has faster growth rate, and more tolerance to high-temperature, and high-light compared with *Synechococcus* *elongatus* PCC 7942, although their genomes are very similar with 99.8% identity (Mueller et al., 2017; Ungerer et al., 2018). The doubling time of Syn2973 can reach 1.9 h in BG11 medium at 41°C under continuous 500 μmol photons m^−2^ s^−1^ white light with 3% CO_2_ (Yu et al., 2015), which was close to that of *S. cerevisiae* (1.67 h) (Herskowitz, 1988). Recently, a series of studies have revealed the mechanism related to the faster growth of Syn2973 than *S. elongatus* PCC 7942 from the genomic, transcriptomic, proteomic, and fluxomic levels under different culture conditions, respectively (Abernathy et al., 2017; Tan et al., 2018; Ungerer et al., 2018; Hendry et al., 2019). In addition, the genetic toolboxes have also been developed and optimized for Syn2973, which greatly facilitated the genetic engineering and synthetic biology for Syn2973 in future (Wendt et al., 2016; Li et al., 2018). Furthermore, Syn2973 has been successfully used for bioproduction, such as sucrose and hapalindole alkaloids, demonstrating that it is a promising candidate to serve as a photosynthetic cell factory (Song et al., 2016; Knoot et al., 2019).

Large-scale cultivation of cyanobacteria is necessary for industrial biotechnological applications and has been investigated in the past (Silkina et al., 2019). In addition, another advantage of large-scale cultivation of cyanobacteria compared with the heterotrophic microalgae was reducing greenhouse gas emissions and decreasing dependence on petroleum-based products, which will greatly promote sustainable and renewable development; however, large-scale cultivation of cyanobacteria involves significant usage of water (Pathak et al., 2018), and will be more practical if it can be conducted using seawater. It is well known that the salinity of the seawater changes from around 3.2 to 4.0%, furthermore, about 55 and 31% of the salt content are Na^+^ and Cl^−^, respectively. High concentration of salts imposes significant abiotic stress as it causes detrimental effects on cyanobacteria growth due to both effects of ionic stress and osmotic pressure (Pade and Hagemann, 2014). Syn2973, as a freshwater cyanobacterium, was sensitive to salt stress, and high concentration of salt significantly inhibited the growth of Syn2973 (Song et al., 2016). It is therefore urgent to improve the salt tolerance of Syn2973 for the future application of this promising chassis.

Cyanobacteria have developed a set of salt acclimation strategies during their long-term evolution, mainly involving two major aspects: the active export of ions and the accumulation of compatible solutes (Pade and Hagemann, 2014; Li et al., 2016). Notably, the export of Na^+^ is one of the fastest processes in salt-shocked cells. Fast Na^+^ export is based on the activation of pre-existing ion-transport systems (Pade and Hagemann, 2014). A series of salt-tolerance relevant transporters have been identified and characterized in cyanobacteria, such as different NhaS antiporters (Elanskaya et al., 2002; Tsunekawa et al., 2009), Mrp (Multiple resistance and pH) antiporter (Blanco-Rivero et al., 2005; Fukaya et al., 2009; Ito et al., 2017; Mormile et al., 2019), and Na^+^-pumping ATPase (Soontharapirakkul et al., 2011). For instance, overexpression of the *nhaP* gene from *Aphanothece halophytica*, encoding a Na^+^/H^+^ antiporter in *S. elongatus* PCC 7942 successfully improved its survival and growth under 0.5 M NaCl condition (Waditee et al., 2002). However, up to now, systematical studies aiming to explore the effect of different kinds of transporters in cyanobacteria are still lacking.

Metabolomics have been demonstrated to be a powerful tool to decipher the relative mechanisms of stress tolerance in various cyanobacteria and microalgae (Li et al., 2016; Aikawa et al., 2019). For example, metabolomics analysis was used for exploring the possible mechanisms of salt tolerance mediated by a putative magnesium transporter Slr1216 (Li et al., 2016). In addition, biomass composition analysis also plays an important role in exploring the mechanism of stress tolerance. For example, the accumulation of carbon reserves, such as glycogen in cyanobacteria and starch and lipids in plants and eukaryotic algae, is also a common mechanism of adaptation to variations in salt, light and nutrient availability (Cano et al., 2018).

In this study, aiming at improving salt tolerance of Syn2973, we cloned and individually overexpressed 21 exogenous putative transporters in Syn2973, and analyzed the salt tolerance of the engineered strains as well as the control strain under 0.4 M NaCl condition. In addition, the metabolomics and biomass composition analyses of parent and genetic strains were performed to investigate the salt tolerance mechanisms. Our findings provide a reference to genetic engineering of Syn2973 for improving salt tolerance and could be valuable for understanding the mechanisms of salt tolerance in cyanobacteria.

## Materials and Methods

### Bacterial Growth Conditions

The wild-type Syn2973 and engineered strains were grown on BG11 agar plate or in BG11 medium (pH 7.5) under a light intensity of ~200 μmol photons m^−2^ s^−1^ in an illuminating or shaking incubator of 130 rpm at 37°C (HNYC-202T, Honour, Tianjin, China) (Li et al., 2018). Appropriately antibiotic, 20 μg/mL spectinomycin (Solarbio, Beijing, China) was added to maintain the stability of the engineered strains. Cell optical density was measured by a spectrophotometer (UV-1750, Shimadzu, Kyoto, Japan) at 750 nm. The pH of each culture was measured by a pH meter (FE20, Mettler-Toledo, Zurich, Switzerland). *E. coli* DH5α was grown on LB agar plate or in LB liquid medium in the incubator at 37°C or shaking incubator at 200 rpm supplemented with 50 μg/mL spectinomycin or 200 μg/mL ampicillin (Solarbio, Beijing, China).

### Strains and Plasmids Construction

Strains and plasmids used in this study are listed in Table S1. Among them, *E. coli* DH5α was used for vector construction and amplification. For genes overexpression, an integrative vector pSI with spectinomycin-resistant cassette was used (Li et al., 2018). Primers for gene overexpression are listed in Table S2. Target genes were ligated into pSI plasmids by blunt end connection. The constructed plasmid was finally transformed into Syn2973 according to the method reported previously (Li et al., 2018). Briefly, *E. coli* HB101 and *E. coli* DH5α harboring target plasmid were cultured overnight and then transferred into fresh LB medium with 50 μg/mL spectinomycin or 200 μg/mL ampicillin (Solarbio, Beijing, China) at 1:50 ratio. When cells grew to exponential phase (OD_600_ ≈ 0.5), 2 mL of each *E. coli* strain was washed twice by fresh (LB) medium to remove all the antibiotics, then re-suspended in 0.1 mL of the LB medium, mixed together, and incubated for 30 min. 1 mL of the exponentially growing Syn2973 (OD_750_ ≈ 1) culture was centrifuged and re-suspended in 0.2 mL BG11 medium for each conjugation. The sample was then mixed with the *E. coli* suspension mentioned above and incubated for 30 min. The mixtures were spread on sterile filters (0.45 μm pore size) coated on the BG11 agar plates. After incubated for 24 h at intensity of ~100 μmol photons m^−2^ s^−1^, the filter was transferred onto a new BG11 agar plates with 50 μg/mL spectinomycin. Clones would be seen after incubated at intensity of ~200 μmol photons m^−2^ s^−1^ for about 5 days.

### Neighbor-joining Phylogenetic Tree Construction and Multiple Sequence Alignment

The neighbor-joining phylogenetic tree was constructed by MEGA 5.1 using the default alignment parameters (Tamura et al., 2011). Bootstrap values calculated from 1,000 trees were shown at each node. Multiple sequence alignment was performed using ClustalW software (https://www.genome.jp/tools-bin/clustalw).

### Extraction and Measurement of the Metabolome

Samples (8 mL) of mid-exponential cultures (48 h), at the OD_750_ of 1 ± 0.1, were rapidly harvested by centrifugation at 8,000 × *g* for 8 min at 25°C (Eppendorf 5430R, Hamburg, Germany). The extraction of metabolites was carried out as previously published with slight modification (Bennette et al., 2011; Wang et al., 2014). ^13^C3, ^15^N-alanine (Cambridge Isotope Laboratories, Inc., Andover, MA, USA) was added as the internal standard to correct for variation due to sample extraction and injection. Briefly, roughly 8 OD_750_ of cells were added with 900 μL of the solution containing Methanol/H_2_O (8:2, *v*/*v*), and then frozen-thrawed for three times. Samples were centrifuged at 15,000 × *g* for 5 min at 4°C. The supernatant was extracted and the sediment was repeated the above extraction process. The supernatants were mixed and the solvents were removed using a vacuum concentrator system (ZLS-1, Her-exi, Hunan, China). For LC-MS analysis, each dried sample was dissolved in 100 μL of purified water. For GC-MS analysis, each sample was further derivatized in two steps as previously published (Cui et al., 2016).

Sugar phosphates and AcCoA were measured by LC-MS analysis. The LC-MS analysis was conducted on an Agilent 1260 series binary HPLC system (Agilent Technologies, Santa Clara, CA, USA) using a XBridge Amide column (150 × 2.1 mm, 3.5 μm; Waters, Milford, MA, USA), coupled to an Agilent 6410 550 triple quadrupole mass analyser equipped with an electrospray ionization source (ESI). The multiple reaction monitoring (MRM) mode was used for scanning. The MRM pairs of the standard metabolites were AcCoA (ESI+, 809.9 → 303), ^13^C3, ^15^N-alanine (ESI+, 94 → 47), PEP (ESI-, 166.9 → 79), NADPH (ESI-, 744 → 159), ATP (ESI-, 426 → 134), ADP-GLC (ESI-, 588 → 346), UDP-GLC (ESI-, 565 → 323), FBP (ESI-, 339 → 97), R5P (ESI-, 229 → 97), E4P (ESI-, 199.1 → 97), RU5P (ESI-, 309 → 97), G6P/F6P (ESI-, 259 → 97), GAP (ESI-, 169 → 97), 2/3PG (ESI-, 185 → 79), MAL (ESI-, 133 → 115), FUM (ESI-, 115 → 71), AKG (ESI-, 145 → 101). All of the peaks were integrated by Qualitative Analysis B.06.00 software and Xcalibur (version 2.1) (Niu et al., 2015). Sucrose and carboxylic acids were derivatized and analyzed on Agilent 5975B/6890N GC-MS instrument (Agilent Technologies, Santa Clara, CA, USA) as previously described (Cui et al., 2016). The metabolites data were normalized by internal standard and cell number. Glycogen extraction and determination were followed by the method described by Song et al. (2016).

### Biomass Composition Analysis

To determine the macromolecular composition of strains JY00 and JY13 under 0 M and 0.4 M NaCl conditions, Briefly, the cells were collected at mid-exponential cultures (48 h), at the OD_750_ of 1 ± 0.1, by centrifugation (3,550 × g) for 5 min and freeze-dried to generate a lyophilized powder. The Lowry method (Holdsworth et al., 1988) was used to measure protein content. The phenol-sulfuric acid method was used to determine intracellular carbohydrate contents (Masuko et al., 2005). The total lipids were extracted using a previous method as described below (Cui et al., 2018). Briefly, 50 mg of lyophilized algal powder was used for extraction using a chloroform-methanol solution (2:1, v/v) with 0.01% butylated hydroxytoluene. The extraction process was repeated three to four times. The above extracts were washed with 1.0 mL of 1.0 M KCl followed by 1.0 mL of double-distilled water. The solvents were removed using a vacuum concentrator system (ZLS-1, Her-exi, Hunan, China).

### Lipid Profile Analysis

Cells of strains JY00 and JY13 under 0 M and 0.4 M NaCl conditions were rapidly harvested in mid-exponential cultures (48 h), at the OD_750_ of 1 ± 0.1, by centrifugation at 8,000 × *g* for 8 min at 25°C (Eppendorf 5430R, Hamburg, Germany), and freeze-dried to generate a lyophilized powder. The lipid profile was analyzed according to a previous publication (Xiong et al., 2008). Briefly, 50 mg dry cells were suspended in a mixture of 2 mL methanol acidified with 3% sulfuric acid and 2 mL chloroform containing 2.5 g/L heptadecanoic acid (the internal standard to correct transesterification and injection volume errors). The mixture was then heated in a sealed tube at 100°C for 4 h. After cooling, 1 mL of distilled water was added and the sample was vortexed for 20 s. GC-MS experiments were performed using used a modified method described previously (Cui et al., 2018).

### Statistical Analysis

In this study, each experiment was performed in three biological replicates. All data were reported as means ± standard deviations. A statistical *t*-test model was applied for the comparative analysis, and a *p* < 0.05 was considered statistically significant.

## Results and Discussions

### Poor Salt Tolerance of Syn2973

**Figure 1:**
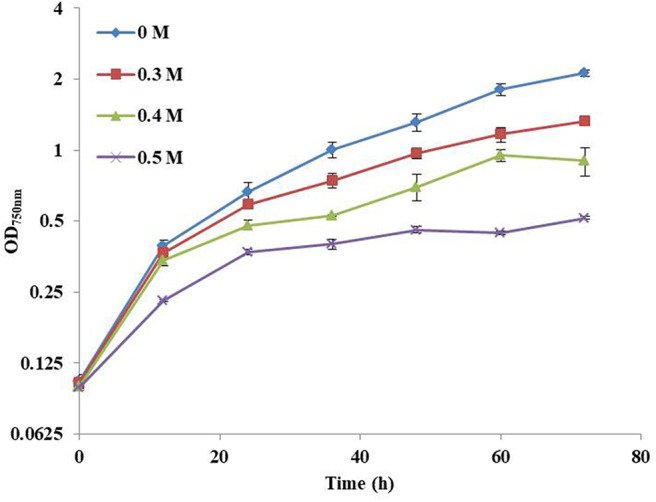
Growth curves of Syn2973 in BG-11 medium under different concentrations of NaCl (0 M, 0.3 M, 0.4 M, and 0.5 M).

In this study, the influence of NaCl concentration on the growth of Syn2973 was first investigated. As shown in Figure 1, the growth of Syn2973 was inhibited by ~23.9, 46.7, and 64.8% at the cultivation time point of 48 h under 0.3, 0.4, and 0.5 M NaCl conditions, respectively, suggesting that the salt tolerance of Syn2973 was significantly lower than other model cyanobacteria such as *Synechocystis* sp. PCC 6803, the growth of which was decreased by 50% under 0.8 M NaCl (Qiao et al., 2013).

### Improved Salt Tolerance of Syn2973 by Screening 21 Exogenous Putative Transporters

**Figure 2:**
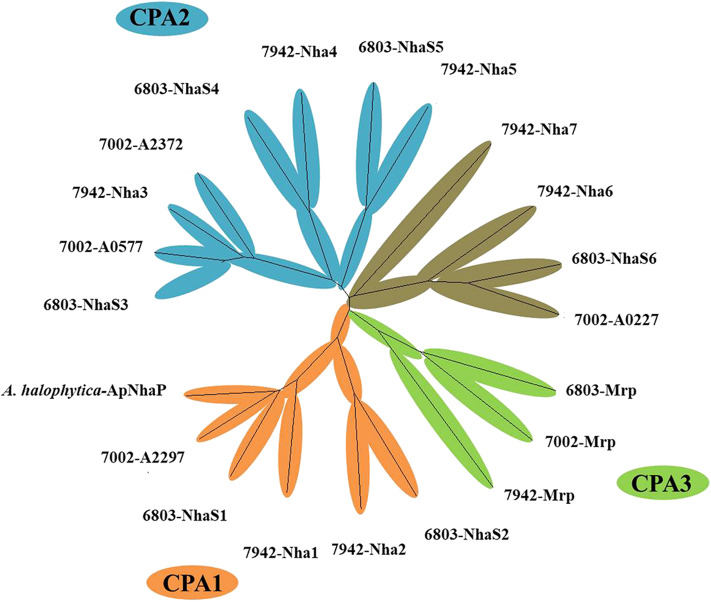
Neighbor-joining phylogenetic tree of transporter proteins from high, moderate, and poor salt tolerant cyanobacteria.

A range of transporters have been demonstrated to play key roles in stress tolerance, such as the ATP-binding cassette (ABC) transporter involved in acid stress tolerance, the KdpA/KtrB related to osmotic and salt stress tolerance in cyanobacteria (Mukhopadhyay, 2015; Tahara et al., 2015; Checchetto et al., 2016). In this study, different transporters were selected and overexpressed individually in Syn2973 to evaluate their potential effects on salt tolerance. A library containing 21 exogenous putative transporters from halophilic strain *Synechococcus* sp. PCC 7002 and *A. halophytica*, moderate salt tolerance strain *Synechocystis* sp. PCC 6803, and freshwater strain *S. elongatus* PCC 7942 was constructed. The cation/proton antiporters (CPA) families were the main Na^+^/H^+^ antiporter in cyanobacteria (Billini et al., 2008). According to the sequence-based Transporter Classification System, the selected 21 putative transporters here can be divided into CPA1, CPA2, and CPA3 families. The CPA1 and CPA2 families are most single hydrophobic gene products, some of which function as homooligomers. In contrast, the CPA3 (Mrp-type) family are the most structurally complex antiporters, which have six to seven different hydrophobic proteins (Krulwich et al., 2009). As shown in Figure 2, six transporter proteins (*A. halophytica*-ApNhaP, 6803-NhaS1, 7942-Nha1, 6803-NhaS2, 7942-Nha2, and 7002-A2297) belong to the CPA1 family, eight transporter proteins (7002-A0577, 6803-NhaS3, 7942-Nha3, 7002-A2372, 7942-Nha4, 6803-NhaS4, 7942-Nha5, and 6803-NhaS5) belong to the CPA2 family, and three transporter proteins (6803-Mrp, 7002-Mrp, and 7942-Mrp) belong to the CPA3 family. Moreover, a number of other transporters (7942-Nha7, 7942-Nha6, 6803-NhaS6, and 7002-A0227) were also selected for evaluation.

**Figure 3:**
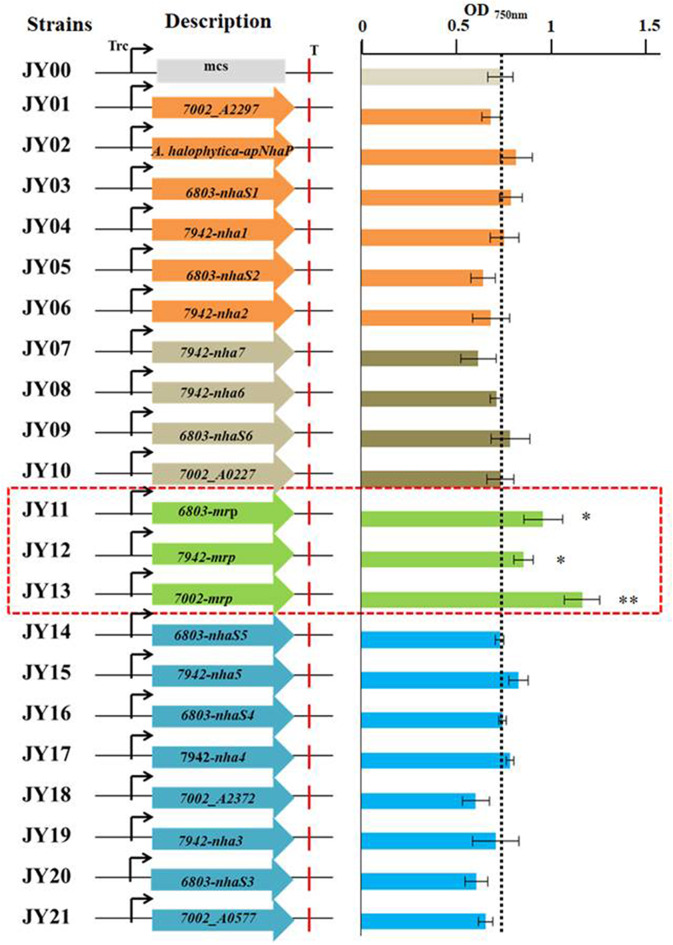
The values of OD_750_ of all the engineered strains at the cultivation time point of 60 h under 0.4 M NaCl.

In this study, the 21 putative transporters were overexpressed individually in Syn2973 under the control of the strong promoter of P_*trc*_. Among them, Mrp transporter including six to seven proteins were all overexpressed together. Then the salt tolerance of the engineered strains as well as the control strain (strain JY00) was analyzed in BG-11 medium under 0.4 M NaCl condition. The values of OD_750_ of the strains at cultivation time point of 60 h were shown in Figure 3, and the results showed that the growth of strains JY11 overexpressing 6803-Mrp, JY12 overexpressing 7942-Mrp, and JY13 overexpressing 7002-Mrp was improved by 30.3, 16.5, and 57.7% respectively, compared with the control strain JY00, suggesting that the overexpression of the 6803-Mrp, 7002-Mrp, and 7942-Mrp transporters from the CPA3 family (Mrp-type) could improve the salt tolerance of Syn2973. Notably, the OD_750_ of strain JY13 overexpressing the Mrp antiporter from *Synechococcus* sp. PCC 7002 was significantly improved by 57.7% under 0.4 M NaCl condition, while no significant change was observed for its growth compared with strain JY00 without NaCl (Figure S1).

Mrp antiporters are multisubunit operon systems that catalyze the efflux of monovalent cations and facilitate the influx of protons (Swartz et al., 2005). It had been reported that the Mrp antiporters took part in Na^+^ resistance in *Bacillus subtilis* (Ito et al., 1999). Previous study had shown that the growth of engineered *S. elongatus* PCC 7942 with *mrpA* disruption was severely inhibited when the cells were grown at high salinity, which suggested that the Mrp transporter might play an important role in Na^+^ resistance in cyanobacteria (Fukaya et al., 2009). Another research showed that Mrp antiporters played key roles in high alkaline stress in the alkaline- and salt-tolerant *Dietzia* sp. DQ12-45-1b (Fang et al., 2018). Recently, a study showed that the expression of Mrp-systems were up-regulated in marine cyanobacteria *Euhalothece* sp. Z-M001 under salt stress, suggesting their importance in hypersaline habitats (Yang et al., 2020). Similar phenomena were confirmed in our study. It is worth noting that the three Mrp antiporters exhibited different effect on Na^+^ resistance in this study, although they shared high sequence identity (>55%). The Mrp antiporter from *Synechococcus* sp. PCC 7002 performed the best, while the Mrp antiporter from *S. elongatus* PCC 7942 showed the least effect. The Mrp A from *Bacillus pseudofirmus* OF4 has undergone intensively functional and structural analyses (Ito et al., 2017). The conserved residues which involved in antiport activity have been thoroughly studied (Morino et al., 2010). Sequence alignment showed that 6803-Mrp, 7002-Mrp, and 7942-Mrp shared homolog with Mrp A (Figure S2). It was reported that Mrp A would lose the Na^+^/H^+^ antiport activity if lysine at 299 site was mutated to alanine or arginine at 773 site was mutated into alanine (Ito et al., 2017). For 7002-Mrp and 6803-Mrp, the corresponding sites were lysine and arginine. However, for 7942-Mrp, they were isoleucine and glycine. Isoleucine and glycine share similar features with alanine. These might explain why strain JY12 overexpressing 7942-Mrp demonstrated the least effect on the improvement of salt tolerance. It is an interesting hypothesis, which still needs to be intensively investigated in further.

CPA1 family 6803-NhaS1, CPA2 family 6803-NhaS3, and CPA2 family 6803-NhaS4 were active Na^+^/H^+^ antiporters involved in Na^+^ antiporting. Among them, 6803-NhaS3 showed the highest transport activity than the 6803-NhaS1, 6803-NhaS4, which probably performed majority of Na^+^ exporting under saline conditions (Inaba et al., 2001). Previous study showed that the CPA1 family *A. halophytica*-ApNhaP exhibited high Na^+^/H^+^ exchange activity over a wide range of pH with novel ion specificity (Waditee et al., 2001). In addition, a previous study showed that of the putative 7942-Nha transporters expressed in *E. coli*, only the CPA2 family 7942-Nha3 complemented the deficient Na^+^/H^+^ antiporter activity of the Na^+^-sensitive *E. coli* TO114 strain (Billini et al., 2008). However, in this study, we found that the 6803-NhaS3, 7942-Nha3 and *A. halophytica*-ApNhaP played little role in the salt resistance of Syn2973. The possible reasons may be that they are irrelevant to Na^+^ transportation or the expression level of these transporters has already saturated in Syn2973 even if they are involved in Na^+^ extrusion. These should be further investigated in future.

It was well known that Na^+^/H^+^ antiporter extruded Na^+^ in exchange for H^+^ (Tsunekawa et al., 2009). In addition, Na^+^/H^+^ antiporters are important for Na^+^ resistance as well as for pH homeostasis (Fukaya et al., 2009). Mrp antiporters belonged to Na^+^/H^+^ antiporter, which might be associated with pH regulation. Thus, the extracellular pH of strains JY00 and JY13 under 0.4 M NaCl conditions were determined (Figure S3). The results showed the pH increased from ~7.5 to ~10.5 with the increase of cell density and culture time in both strains JY00 and JY13 under 0.4 M NaCl conditions. It was consistent with the previous study that alkaline pH might favor the formation of bicarbonate and promoted the cyanobacteria cell growth (Blanco-Rivero et al., 2005). Besides, the pH of strain JY13 was slightly higher ~5% than strain JY00 at cultivation time point of 36 h, suggesting that overexpressing 7002-Mrp might affect pH by Na^+^/H^+^ exchange. However, the pH didn't show significantly differences between strains JY00 and JY13 across the growth profile. It was possibly that the pH was dynamic during cyanobacteria growth, which was influenced by different factors, such as the bicarbonate concentration, H^+^-pumping ATPase, and other H^+^-related transporters.

### Comparative Targeted Metabolomics Analysis of Strains JY13 and JY00

In addition to Na^+^ extrusion, additional mechanisms relevant to improved salt tolerance in strains JY00 and JY13 were comparatively explored. The metabolites of strains JY00 and strain JY13 under 0 and 0.4 M NaCl conditions were extracted and subjected to LC- and GC-MS based targeted metabolomics analysis. All the data were normalized by the value of the strain JY00 under 0 M NaCl condition (Table 1).

**Table 1:** Comparison of the metabolites between the strains JY00 and JY13 under 0 M and 0.4 M NaCl conditions^*^.

**Metabolites**	**JY13-0 M NaCl**	**JY00-0.4 M NaCl**	**JY13-0.4 M NaCl**
GAP	0.70 ± 0.11	6.78 ± 0.09	8.58 ± 1.97
2/3 PG	0.52 ± 0.09	2.72 ± 0.34	0.74 ± 0.09
FBP	1.28 ± 0.09	6.52 ± 0.78	14.38 ± 1.69
PEP	0.82 ± 0.17	1.26 ± 0.09	0.63 ± 0.10
F6P/G6P	0.87 ± 0.12	7.06 ± 0.21	3.43 ± 0.39
Sucrose	1.52 ± 0.25	22.20 ± 2,01	36.70 ± 3.71
Glycogen	1.44 ± 0.18	1.45 ± 0.15	2.15 ± 0.05
ADP-GLC	0.71 ± 0.08	0.36 ± 0.07	1.03 ± 0.17
UDP-GLC	0.62 ± 0.05	0.24 ± 0.02	0.28 ± 0.02
RU5P	0.97 ± 0.11	2.15 ± 0.11	1.32 ± 0.18
R5P	1.01 ± 0.18	5.38 ± 1.01	5.26 ± 0.58
E4P	0.79 ± 0.18	6.71 ± 1.35	5.79 ± 0.98
AcCoA	1.73 ± 0.27	1.65 ± 0.24	4.09 ± 0.32
CIT	0.72 ± 0.04	1.36 ± 0.11	0.52 ± 0.03
AKG	0.62 ± 0.05	0.58 ± 0.07	0.63 ± 0.11
FUM	0.93 ± 0.18	2.87 ± 0.21	2.40 ± 0.45
MAL	0.74 ± 0.07	1.59 ± 0.26	0.54 ± 0.09
NADPH	1.27 ± 0.11	0.19 ± 0.03	0.64 ± 0.09
ATP	1.25 ± 0.25	1.26 ± 0.17	1.86 ± 0.25

* *The numerical values were the ratio of the abundance of the strain JY13 under 0 M NaCl, strain JY00 under 0.4 M NaCl, strain JY13 under 0.4 M NaCl, to the strain JY00 under 0 M, respectively. The average value for the strain JY00 was set to one. Data show the mean with error bars indicating standard deviation calculated from three independent biological replicates*.

**Figure 4:**
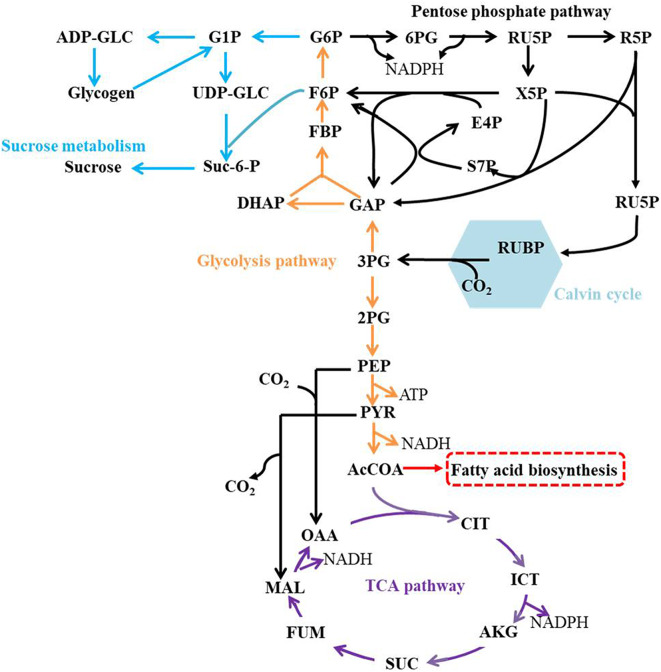
The central metabolic pathways in Syn2973.

The central metabolic pathways in Syn2973 were shown in Figure 4, our analysis found that: (i) for the glycolysis pathway, the content of GAP was significantly increased by 6.78-fold and 8.58-fold in strains JY00 and JY13, respectively, upon the addition of 0.4 M NaCl. In addition, GAP content was increased by 26.5% in strain JY13 with 0.4 M NaCl stress, compared with strain JY00 with 0.4 M NaCl stress. The FBP content was significantly improved by 6.52-fold and 14.38-fold in strains JY00 and JY13 upon salt stress, respectively. The FBP content of strain JY13 under salt stress was elevated by 1.2-fold in comparison with that of strain JY00 under salt stress. Meanwhile, under salt stress, the sucrose was increased by 22.20-fold and 36.70-fold in strains JY00 and JY13, respectively. The sucrose content of strain JY13 with 0.4 M NaCl addition was improved by 65.3%, compared with that of strain JY00 with 0.4 M NaCl addition, which is consistent with the previous study that the sucrose as the compatible solute was induced remarkably under salt stress in Syn2973 (Song et al., 2016). The glycogen content was elevated by 44.7 and 49.4% in strains JY00 and JY13, respectively, with 0.4 M NaCl addition. And the glycogen content of strain JY13 with 0.4 M NaCl addition was increased by 48.3% in comparison with that of strain JY00 with 0.4 M NaCl addition. Glycogen plays important physiological roles in cyanobacteria to maintain homeostasis and resist environmental stresses (Luan et al., 2019). Our results suggested that the flux might be redirected from the glycolytic pathway to sucrose and glycogen biosynthesis for enhancing salt resistance in Syn2973, and overexpressing 7002-Mrp further strengthened this process. However, UDP-GLC, the precursor of sucrose, was decreased by ~80% under salt stress in both strains, indicating that it might be a bottleneck for hindering flux from G6P to sucrose, which may be target for improving the salt tolerance of Syn2973 in the future. (ii) For the pentose phosphate pathway, the RU5P, R5P, and E4P were enhanced in both strains with 0.4 M NaCl addition. The abundance of these compounds was roughly the same in two strains with 0.4 M NaCl addition. The pentose phosphate pathway belongs to the carbon fixation pathway in *S*. *elongatus*. It was reported that the transcript abundance of carbon fixation was increased in *Prochlorococcus* AS9601 under salt stress (Al-Hosani et al., 2015). The results suggested that Syn2973 elevated pentose phosphate pathway, which might fix more carbon dioxide for salt tolerance, and overexpressing 7002-Mrp has no inference on the mechanism. (iii) For the TCA cycle, the contents of CIT, MAL, and FUM were significantly increased in strain JY00 upon 0.4 M NaCl addition, while they were obviously decreased in strain JY13 upon 0.4 M NaCl addition. Furthermore, the CIT, MAL, and FUM contents were decreased in strain JY13 with 0.4 M NaCl addition, compared with those in strain JY00 with 0.4 M NaCl addition. It was reported that the TCA cycle was enhanced for generating more ATP against salt stress in common wild soybean, while the TCA cycle was attenuated to synthesize more amino acids and organic acids, suggesting that salt-tolerant soybean might regulate amino acid and organic acid metabolism to generate more compatible solutes for resisting salt stress in salt-tolerant soybean (Yang et al., 2017). In our study, the role of TCA cycle on salt tolerance might also be different between common and salt-tolerant Syn2973, and overexpressing 7002-Mrp might reduce TCA activity for generating more compatible solutes to enhance salt tolerance in Syn2973. (iv) For energy metabolites, the contents of NADPH and ATP were decreased in strain JY00 with salt stress, while they were both increased in strain JY13 with salt stress. In addition, NADPH and ATP contents were increased by 3.36-fold and 1.47-fold in strain JY13 with salt condition, compared with strain JY00 with salt condition. Previous researches have revealed that the Mrp antiporter is homologous to the proton transport subunit of the respiratory chain complex I and also takes part in energy metabolism (Blanco-Rivero et al., 2005, 2009; Yu et al., 2018). It was discovered that the energy metabolism was associated with the membrane permeability, which could regulate the membrane assembly and allow ion to move across the membrane by specific transporter (Qi et al., 2019). Therefore, it was reasonably inferred that the energy metabolites might directly promote the efficiency of Mrp antiporter by enhancing membrane permeability. These suggested that energy metabolites might play different roles in the salt tolerance mechanisms between common and salt-tolerant Syn2973.

Together, our results suggested that strain JY00 and strain JY13 shared the similar salt tolerance mechanism, but still existed certain distinct characteristics. Both of them redirected flux from the glycolytic pathway to sucrose and glycogen biosynthesis and fixed more carbon source by elevating the pentose phosphate pathway under salt condition. In addition, overexpressing 7002-Mrp reduced TCA, strengthened more metabolic flux flowing into sucrose and glycogen biosynthesis and elevated NADPH and ATP content, which might be associated with Mrp antiporter efficiency. These collectively improved the salt tolerance of Syn2973.

### Biomass Composition Analysis of Strains JY00 and JY13 Under 0 M and 0.4 M NaCl Conditions

It was discovered AcCoA was significantly increased by 2.4-fold in strain JY13, compared with strain JY00 under salt stress. AcCoA is the precursors of lipid and fatty acids in Syn 2973, whose role in stress tolerance of cyanobacteria and microalga has been well-documented (Singh et al., 2002). To further decipher salt stress tolerance mechanisms, the biomass composition of strains JY13 and JY00 under 0 and 0.4 M NaCl conditions was investigated.

The main components of Syn2973 were carbohydrates, proteins, and lipids. As shown in Table 2, the contents of carbohydrates were significantly increased by 25.8 and 30.3% in strains JY00 and JY13 upon 0.4 M NaCl addition, respectively. In addition, the carbohydrate content of strain JY13 under salt stress was 10.2% higher than that of strain JY00 under salt stress, consistent with the changes of sucrose and glycogen detected by metabolomics analysis. Previous study revealed that the contents of carbohydrates increased ~5-fold in *S. elongates* PCC 7942 under 0.5 M NaCl conditions compared to control (Verma et al., 2018). Thus, it's reasonable to suppose that the increased carbohydrates contents might be used as an adaptive strategy of cyanobacteria under saline conditions. The proteins content of strains JY00 and JY13 were both significantly decreased upon 0.4 M salt addition, and decreased extent was roughly the same between strain JY00 and JY13. For lipid, the contents were increased by 18.9 and 20.6% in strains JY00 and JY13 upon 0.4 M NaCl addition, respectively. Furthermore, the lipid content of strain JY13 with salt stress was higher than that of strain JY00 with salt stress. Previous studies showed that lipid production was enhanced in *Chlamydomonas reinhardtii* under salt stress (Atikij et al., 2019). Similar phenomena were observed in this study. In addition, the relative distribution of fatty acid profiles of strains JY00 and JY13 under 0 and 0.4 M NaCl conditions were also determined (Table 3). The main fatty acids were consisted of C14:0, C14:1, C16:0, C16:1, C18:0, and C18:1, which was consistent with the previous studies (Abernathy et al., 2017). Previous study showed that the unsaturated fatty acid in membrane lipids could protect the photosynthetic machinery against salt-induced damage in *Synechococcus* (Allakhverdiev et al., 2001). In this study, the unsaturated fatty acid content had not significantly changed in JY00 strain, and had slightly improved by 8.07% in JY13 strain under 0.4 M NaCl conditions, respectively. Interestingly, for unsaturated fatty acid composition, the percentage of C16:1 was both decreased in strains JY00 and JY13 after addition of 0.4 M NaCl. However, the percentage of C18:1 was significantly increased by 90% and 3-fold in strains JY00 and JY13, respectively, under salt stress conditions. Furthermore, the percentage of C16:1 in strain JY13 with salt stress was 28.5% lower than that in strain JY00 with salt stress; the percentage of C18:1 in strain JY13 with salt stress was 1.53-fold higher than that in strain JY00 with salt stress. A previous study also showed that the content of C16:1 had significantly decreased by 29.1%, meanwhile, C18:1 increased ~2-fold under 0.5 M NaCl conditions in *S. elongatus* PCC 7942 (Verma et al., 2018). However, the mechanism of conversion between C18:1 and C16:1 under salt stress is still unclear and further research should be performed in the near future.

**Table 2:** Comparison of the biomass composition between the strains JY00 and JY13 under 0 M and 0.4 M NaCl conditions^*^.

**Biomass composition**	**JY00-0 M NaCl**	**JY13-0 M NaCl**	**JY00-0.4 M NaCl**	**JY13-0.4 M NaCl**
Carbohydrate	31.0% ± 0.0273	33.0% ± 0.0297	39.0% ± 0.0351	43.0% ± 0.0387
Protein	37.5% ± 0.0482	36.5% ± 0.0475	29.6% ± 0.0385	29.4% ± 0.0384
Lipid	13.7% ± 0.0163	14.5% ± 0.0174	16.3% ± 0.0196	17.5% ± 0.0212

* *Data were derived from three replicates of cells with strain JY00 under 0 M NaCl conditions, strain JY13 under 0 M NaCl conditions, strain JY00 under 0.4 M NaCl conditions, and JY13 strain under 0.4 M NaCl conditions, respectively*.

**Table 3:** Fatty acid composition in strains JY00 and JY13 under 0 M and 0.4 M NaCl conditions^*^.

**Fatty acids**	**JY00-0 M NaCl**	**JY13-0 M NaCl**	**JY00-0.4 M NaCl**	**JY13-0.4 M NaCl**
C14:1	0.34% ± 0.0004	0.07% ± 0.0001	0.1% ± 0.0001	0.04% ± 0.0001
C14:0	1.69% ± 0.0003	0.61% ± 0.0006	1.31% ± 0.0015	0.5% ± 0.0003
C16:1	35.15% ± 0.0297	31.15% ± 0.0311	25.44% ± 0.0305	18.2% ± 0.0403
C16:0	48.81% ± 0.0211	55.63% ± 0.0556	52.34% ±0.0628	48.35% ± 0.0267
C18:1	8.02% ± 0.0197	7.89% ± 0.0078	15.65% ± 0.0187	24.03% ± 0.0178
C18:0	5.99% ± 0.0313	4.66% ± 0.0046	5.17% ± 0.0061	8.89% ± 0.0318

* *Data were derived from three replicates of cells with strain JY00 under 0 M NaCl conditions, strain JY13 under 0 M NaCl conditions, strain JY00 under 0.4 M NaCl conditions, and JY13 strain under 0.4 M NaCl conditions, respectively*.

Our results suggested that Syn2973 resisted salt stress by elevating the carbohydrates and lipid content, and overexpressing 7002-Mrp further strengthened the process. Furthermore, the genes relevant to lipid biosynthesis and conversion between C18:1 and C16:1 could be potential engineering targets for further increasing salt tolerance of Syn2973.

## Conclusions

In this study, a library containing 21 exogenous putative transporters was constructed and overexpressed in Syn2973 individually. The results showed that the overexpression of three Mrp antiporters from Syn2973 significantly improved the salt tolerance of Syn2973, suggesting that Mrp antiporter played an important role in tolerance to salt stress. Metabolomics and biomass composition analyses showed that the salt tolerance mechanisms of parent and strain overexpressing 7002-Mrp were similar but still have distinct features. Both of them redirected flux from glycolytic pathway to sucrose and glycogen biosynthesis, elevated pentose phosphate pathway possibly for fixing more carbon source and increased lipid content and percentage of C18:1 and decreased the percentage of C16:1 under high salt condition. Overexpressing 7002-Mrp strengthened these processes, except for elevating the pentose phosphate pathway, furthermore, it reduced the TCA cycle to synthesize more amino acids and organic acids and elevated NADPH and ATP content, which might promote Mrp antiporter efficiency. Together, our findings here could be valuable for understanding the mechanisms of salt tolerance in Syn2973 and provide important engineering strategies to further improve salt tolerance in cyanobacteria.

## Data Availability Statement

All datasets generated for this study are included in the article/Supplementary Material.

## Author Contributions

LC and WZ conceived and designed the study. JC performed the experiments. JC, TS, SL, YX, XS, FW, LC, and WZ analyzed the data and wrote the manuscript. All authors read and approved the manuscript.

## Conflict of Interest

The authors declare that the research was conducted in the absence of any commercial or financial relationships that could be construed as a potential conflict of interest.

## Supplementary Material

Figure S1 Growth curves of strains JY00 and JY13 under 0 and 0.4 M NaCl conditions.

Figure S2 Multiple sequence alignment among 6803-Mrp, 7942-Mrp, 7002-Mrp, and *B. pseudofirmus* OF4 Mrp A.

Figure S3 The pH of strains JY00 and JY13 at different cultivation time point under 0.4 M NaCl conditions.

## Abbreviations

2-PG: glycerate 2-phosphate
3-PG: glycerate 3-phosphate
6PG: 6-phospho-gluconate
AcCoA: acetyl-CoA
ADP-GLC: adenosine diphosphoglucose
AKG: a-ketoglutarate
ATP: adenosine triphosphate
CIT: citrate
DHAP: dihydroxyacetone phosphate
E4P: erythrose-4-phosphate
F6P: fructose-6-phosphate
FBP: fructose 1,6-diphosphate
G1P: glucose 1-phosphate
G6P: glucose-6-phosphate
GAP: glyceraldehyde 3-phosphate
ICT: isocitrate
MAL: malate
NADH: nitcotinamide adenine dinucleotide (reduced)
OAA: oxaloacetate
PEP: phosphoenolpyruvate
PYR: pyruvate
R5P: ribose 5-phosphate
Ru5P: ribulose 5-phosphate
RUBP: ribulose 1,5-diphosphate
S7P: seduheptulose-7-phosphate
SUC: succinate
Suc-6-P: sucrose 6-phosphate
UDP-GLC: uridine diphosphate glucose
X5P: xylulose 5-phosphate.
