# Supplementary figures and images for: Improved Salt Tolerance and Metabolomics Analysis of Synechococcus elongatus UTEX 2973 by Overexpressing Mrp Antiporters
Source: Front Bioeng Biotechnol. 2020 May 26;8:500. doi: 10.3389/fbioe.2020.00500 (PMC7264159; doi:10.3389/fbioe.2020.00500)

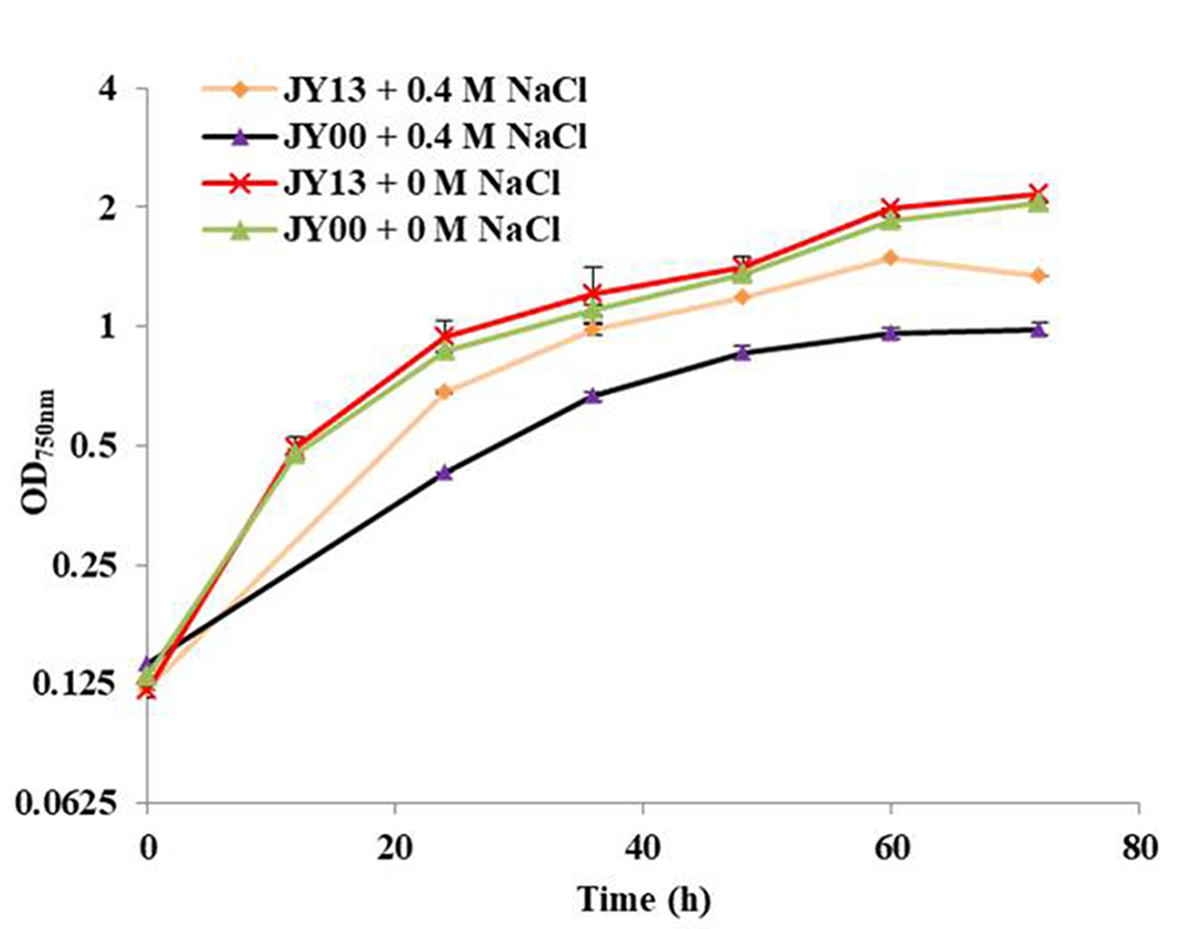

Supplement: Figure S1 — Growth curves of strains JY00 and JY13 under 0 and 0.4 M NaCl conditions. [file Image_1.TIF]

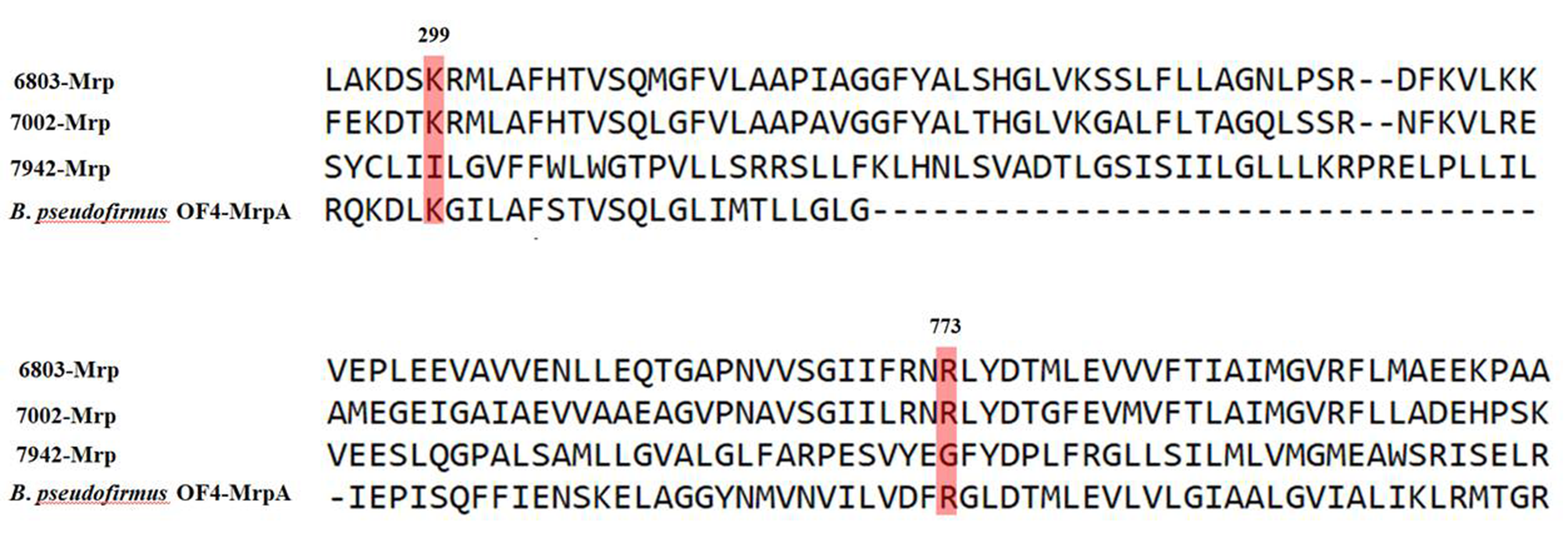

Supplement: Figure S2 — Multiple sequence alignment among 6803-Mrp, 7942-Mrp, 7002-Mrp, and B. pseudofirmus OF4 Mrp A. [file Image_2.TIF]

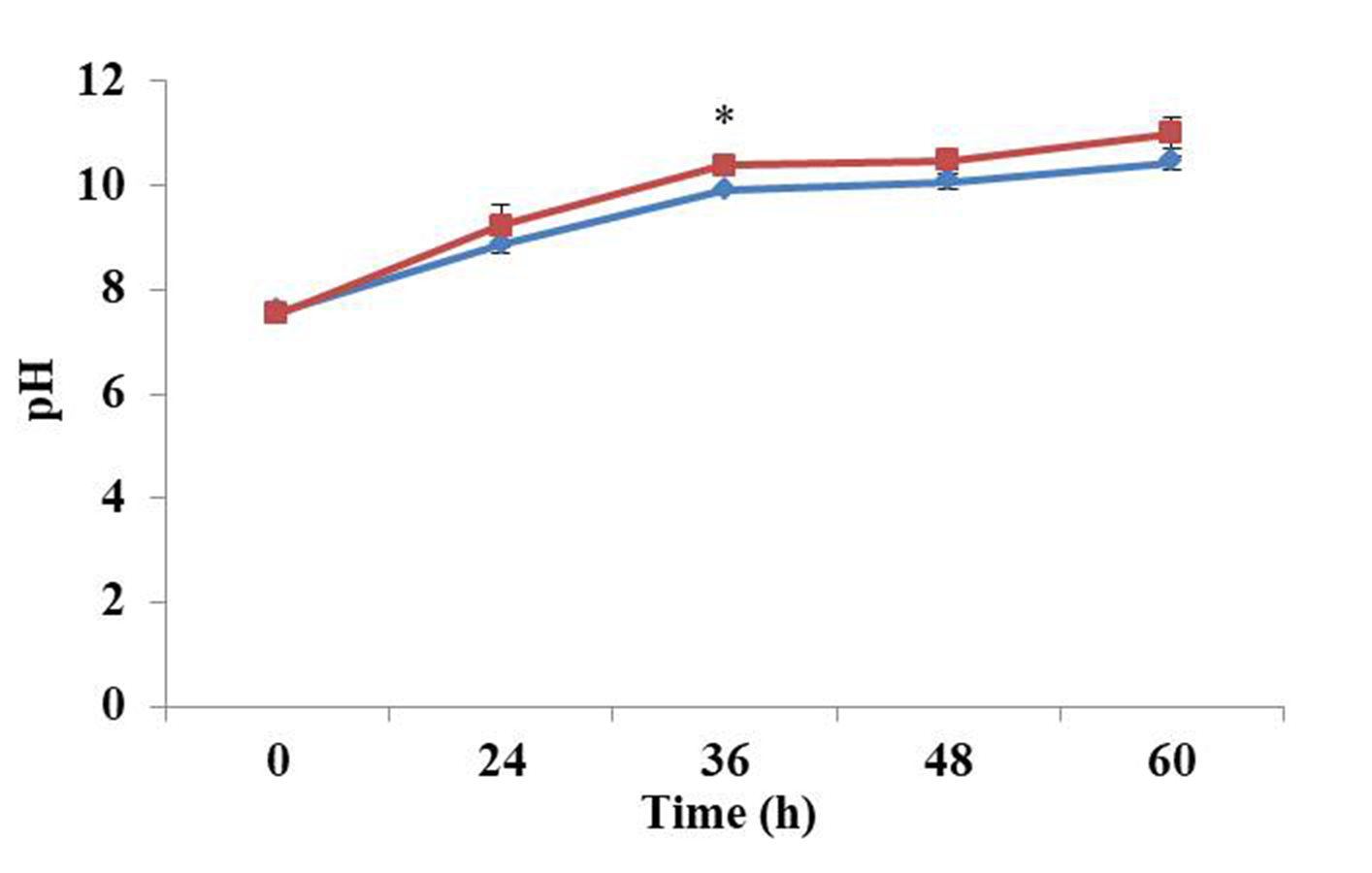

Supplement: Figure S3 — The pH of strains JY00 and JY13 at different cultivation time point under 0.4 M NaCl conditions. [file Image_3.TIF]
