# Supplementary material for: Improved Salt Tolerance and Metabolomics Analysis of Synechococcus elongatus UTEX 2973 by Overexpressing Mrp Antiporters
Source: Front Bioeng Biotechnol. 2020 May 26;8:500. doi: 10.3389/fbioe.2020.00500 (PMC7264159; doi:10.3389/fbioe.2020.00500)
Supplement: Supplementary file 4 [file Table_1.DOCX]

| **Table S1. Strains and plasmids used in this study.** | |  |
| --- | --- | --- |
| Strains/plasmids | Genotype or relevant features | References |
| *E.coli* strains |  |  |
| DH5α | F^-^φH5ao*lacZ*ΔM15 Δ(*lacZYA*-*argF*) U169 *endA*1 *recA*1 *hsdR*17 (r_k_^-^,m_k_^+^) *supE*44, λ^-^ *thi*-1 *gyrA*96 *relA*1 *pho*A | TransGen Biotech |
| HB101 | *supE*44, Δ(*mcrC*-*mrr*), *recA*13, *ara*-14, *proA*2, *lacY*1, *galK*2, *rpsL*20, *xyl*-5, *mtl*-1, *leu*B6, *thi*-1 | Takara Bio |
| Plasmids |  |  |
| pSI | NSI::P*_trc_*-*mcs*-T*_rbcL_*; *spe^R^* | Li et al., 2018 |
| pJY01 | NSI::P*_trc_*-*7002_A2297*-T*_rbcL_*; *spe^R^* | In this study |
| pJY02 | NSI::P*_trc_*- *apNhaP*-T*_rbcL_*; *spe^R^* | In this study |
| pJY03 | NSI::P*_trc_*-*nhaS1*-T*_rbcL_*; *spe^R^* | In this study |
| pJY04 | NSI::P*_trc_*- *nha1*-T*_rbcL_*; *spe^R^* | In this study |
| pJY05 | NSI::P*_trc_*- *nhaS2*-T*_rbcL_*; *spe^R^* | In this study |
| pJY06 | NSI::P*_trc_*- *nha2*-T*_rbcL_*; *spe^R^* | In this study |
| pJY07 | NSI::P*_trc_*-*nha7*-T*_rbcL_*; *spe^R^* | In this study |
| pJY08 | NSI::P*_trc_*- *nha6*-T*_rbcL_*; *spe^R^* | In this study |
| pJY09 | NSI::P*_trc_*- *nhaS6*-T*_rbcL_*; *spe^R^* | In this study |
| pJY10 | NSI::P*_trc_*-*7002_A0227*-T*_rbcL_*; *spe^R^* | In this study |
| pJY11 | NSI::P*_trc_*-*6803-mrp* -T*_rbcL_*; *spe^R^* | In this study |
| pJY12 | NSI::P*_trc_*-*7942-mrp*-T*_rbcL_*; *spe^R^* | In this study |
| pJY13 | NSI::P*_trc_*-*7002-mrp* -T*_rbcL_*; *spe^R^* | In this study |
| pJY14 | NSI::P*_trc_*-*6803-nhaS5* -T*_rbcL_*; *spe^R^* | In this study |
| pJY15 | NSI::P*_trc_*-*6803-nha5* -T*_rbcL_*; *spe^R^* | In this study |
| pJY16 | NSI::P*_trc_*-*6803-nhaS4*-T*_rbcL_*; *spe^R^* | In this study |
| pJY17 | NSI::P*_trc_*-*6803-nha4*-T*_rbcL_*; *spe^R^* | In this study |
| pJY18 | NSI::P*_trc_*-*7002-A2372*-T*_rbcL_*; *spe^R^* | In this study |
| pJY19 | NSI::P*_trc_*-*7942-nha3*-T*_rbcL_*; *spe^R^* | In this study |
| pJY20 | NSI::P*_trc_*- *6803-nhaS3*-T*_rbcL_*; *spe^R^* | In this study |
| pJY21 | NSI::P*_trc_*-*7002_A0577*-T*_rbcL_*; *spe^R^* | In this study |
| Cyanobacteria strains |  |  |
| WT | wild type Syn2973 |  |
| JY00 | pJY00 inWT | In this study |
| JY01 | pJY01 inWT | In this study |
| JY02 | pJY02 inWT | In this study |
| JY03 | pJY03 inWT | In this study |
| JY04 | pJY04 inWT | In this study |
| JY05 | pJY05 inWT | In this study |
| JY06 | pJY06 inWT | In this study |
| JY07 | pJY07 inWT | In this study |
| JY08 | pJY08 inWT | In this study |
| JY09 | pJY09 inWT | In this study |
| JY10 | pJY10 inWT | In this study |
| JY11 | pJY11 inWT | In this study |
| JY12 | pJY12 inWT | In this study |
| JY13 | pJY13 inWT | In this study |
| JY14 | pJY14 inWT | In this study |
| JY15 | pJY15 inWT | In this study |
| JY16 | pJY16 inWT | In this study |
| JY17 | pJY17 inWT | In this study |
| JY18 | pJY18 inWT | In this study |
| JY19 | pJY19 inWT | In this study |
| JY20 | pJY20 inWT | In this study |
| JY21 | pJY22 inWT | In this study |

| **Table S2. Sequence of primers used in this study.** | |
| --- | --- |
| Primers | Sequence (5’–3’) |
| *7002_A2297*-F | ATGCCTTTGGTCATGATTGTTTTAG |
| *7002_A2297*-R | TTAACTCTGAATTGTTTTTTCGGTGG |
| *apNhaP-*F | ATGACCATCGAAGCCGCC |
| *apNhaP-*R | CTATTCCGGTTTCGCTAAAAC |
| *6803-nhaS1*-F | GTGGATACAGCGGTCAACG |
| *6803-nhaS1*-R | CTAGGATGGTTCGGCCAC |
| *7942-nha1*-F | ATGAGCCTTGAAGTCCTGGC |
| *7942-nha1*-R | TTAGTCGGCGATCGCGGG |
| *6803-nhaS2*-F | ATGATTAAGCTCCCTGTGC |
| *6803-nhaS2*-R | TCAGTCATCCTGCAGGGC |
| *7942-nha2*-F | TTGTCGCACACTAGAGGGCG |
| *7942-nha2*-R | TTAGTCCGCTTCAGCCCGCAG |
| *7942-nha3*-F | ATGCTTCAATTCTTGAGTTCAGC |
| *7942-nha3*-R | CTAAGAGCCAGGTTCCACAGCA |
| *7002_A0577*-F | ATGCCGTATTTTCAATCG |
| *7002_A0577*-R | TTATTGTAGCTTCTCCGG |
| *6803-nhaS3*-F | ATGTTTATGAACCCATTGCTCC |
| *6803-nhaS3*-R | CTAATCTGGGGTGGGAACTG |
| *7002_A2372*-F | ATGCTAAGTCCCACAATC |
| *7002_A2372*-R | CTACACCGACAAACTTTTC |
| *7942-nha4*-F | ATGCACTTCAGCCACGTTGTG |
| *7942-nha4*-R | TCATTGAGTGGTGGCAAGCG |
| *6803-nhaS4*-F | ATGGACACCAATACTTTACTG |
| *6803-nhaS4*-R | TTAATGGGCTGGGGCAGG |
| *7942-nha5*-F | ATGCTGTTTGCAGCGATCGC |
| *7942-nha5*-R | TTAATCGAGGGAAGGTACGG |
| *6803-nhaS5*-F | ATGGATGGCCTATTCGCAC |
| *6803-nhaS5*-R | CTAGGCGTAGGGATCGCC |
| *7002_A2373*-*A2380*-F | ATGTCGATGTCTATGCTCGAAG |
| *7002_A2373*-*A2380*-R | CTACAGTAAACCGCGATAGCG |
| *6803-mrp-*F | ATGGTCGCCCTAGAGGCTTGT |
| *6803-mrp -*R | GGTCGATTGGGGTTTTTGGGA |
| *7942-mrp-*F | ATGAAGTGGATTTACCTTGCTG |
| *7942-mrp-*R | TTAGCCGCCAATGGTTTGGGCA |
| *7942-nha7*-F | ATGTTGGCGAATTGGATTGG |
| *7942-nha7*-R | TCAGGAAGTCTGAAGCTCAG |
| *7942-nha6*-F | ATGGATTCTGGACTCGACACC |
| *7942-nha6*-R | TTACGAGCTGGGTGTTGCTG |
| *6803-nhaS6*-F | ATGGAAGGATCATTTTCCC |
| *6803-nhaS6*-R | CTAGTAGATGGGGACTTCTTC |
| *7002_A0227*-F | ATGACAGATTCTTTTGATCTTAC |
| *7002_A0227*-R | TTAACTGTTCTCTGGAAGCTC |
